# Interoception predicts mental imagery vividness: exploring a key relationship

**DOI:** 10.1038/s41598-026-43805-0

**Published:** 2026-03-19

**Authors:** Yoko Nagai, Sofia Arooj, Tamarin R. Futeran-Blake, Ceryn Manders, Hugo Critchley, Juha Silvanto

**Affiliations:** 1 Brighton & Sussex Medical School, Department of Neuroscience, University of Sussex, Brighton, UK; 2 School of Psychology, Faculty of Health and Medical Sciences, University of Surrey, Guildford, UK; 3 Centre for Cognitive and Brain Sciences, University of Macau, Macau, SAR China; 4 Department of Psychology, University of Macau, Macau, SAR China

**Keywords:** Cognitive neuroscience, Emotion, Visual system

## Abstract

Interoception, the sense of the internal state of the body, plays a fundamental role in emotional awareness and self-referential processing. Recently, interoception has been linked to the experience of mental imagery. However, this association has not been empirically characterized. We therefore tested how task-based and self-reported measures of interoception predict individual differences in task-based and self-reported measures of mental imagery. Participants (*N* = 104) completed two heartbeat detection tasks (heartbeat tracking and heartbeat discrimination; assessing objective (behavioural) interoceptive performance accuracy), and the Multidimensional Assessment of Interoceptive Awareness (MAIA) questionnaire, assessing subjective (self-reported) dimensions of interoceptive experience. Behavioural and self-reported aspects of mental imagery were assessed respectively from performance of a mental rotation task and from scores on the Vividness of Visual Imagery Questionnaire (VVIQ). Results revealed that, across participants, interoceptive (heartbeat discrimination performance) accuracy predicted mental rotation ability. In contrast, both heartbeat tracking accuracy and self-reported interoceptive awareness (MAIA) predicted self-reported vividness of mental imagery (VVIQ). Interoceptive measures did not predict performance on a control task (2-back working memory task). Together, these findings suggest that distinct components of interoception underpin different aspects of mental imagery: Heightened (cardioceptive) physiological sensitivity facilitates the active deployment of imagery in mental rotation and enhances the vividness of imagery experience. Moreover, people reporting more subjective sensitivity to interoceptive state also perceive greater vividness of mental imagery. These new results underscore the influence of bodily representation in shaping conscious experience through both controlled and spontaneous expressions of mental simulation.

## Introduction

Interoception is the ability to sense, interpret, and integrate internal bodily signals including heartbeats, respiration, and visceral sensations. Interoception acts as a crucial bridge between bodily physiology and conscious experience^1,2^. While interoception is proximally linked to motivational states, emotional feelings and bodily awareness, it also serves as a substrate for higher-order cognitive functions. The integration of bodily signals with cognitive processes informs decision-making and allows for adaptive responses to uncertainty^2–4^. Individuals with greater interoceptive accuracy typically demonstrate improved error detection and more effective decision-making, particularly in emotionally and socially significant contexts^4^. Furthermore, interoception underpins an integrated representation of a ‘biological self’ from which, arguably, is built a stable, unified sense of self as an active agent^2,5,6^.

The integration of bodily states with both perception and cognition extend to mental imagery, i.e. the ability to generate and manipulate sensory representations in the absence of a direct sensory input^7^. Mental imagery is exemplified by visual representations, yet it is inherently multimodal, involving the integration of emotional and physiological components alongside representations in visual and other sensory modalities^8^. For example, imagining a familiar person extends beyond the sensory recollection of visual features to evoke emotional feelings and bodily states associated with past interactions. From this perspective, interoceptive signals may serve as a physiological anchor for imagery, ensuring that internally generated experiences remain owned and embodied rather than purely abstract (see Muraki et al., 2023^9^, for an embodied perspective on mental imagery).

Consistent with this perspective, self-ratings of interoceptive ‘awareness’ are linked to mental imagery: Individuals who score high for traits of mindfulness (the disposition of persons toward a heightened awareness of multi sensory stimuli) report both more vivid imagery^10,11^. Moreover, individuals with aphantasia, who are unable to experience mental imagery, report lower scores on these same interoceptive dimensions^12^. More recently, we also demonstrated affective disturbances may contribute to imagery loss by modifying the subjective perception of autonomic signals and interfering with the integration of bodily, emotional, and cognitive information necessary for the formation of vivid mental representations^13^.

However, self-reported interoception is not a measure of actual interoceptive ability, i.e. the accurate perception of internal bodily signals. For example, adults with autism often report high subjective interoceptive sensitivity, yet on more behavioural measures, notably heartbeat detection tasks, show significant impairments in task performance^14^. Similarly, scores on the questionnaire ‘Interoceptive Accuracy Scale’, are linked to visual imagery and aphantasia^12,15^, yet there is a poor correlation between self-ratings this scale and behavioral measures of interoceptive task performance accuracy^16^. These discrepancies underscore the importance of including task-based assessments of interoception to characterize how dimensions of interoception, including differences in interoceptive ability, relate to mental imagery.

To address this issue, we employed both task-based and self-reported interoceptive measures to investigate the relationship between interoception and mental imagery phenomenology. Specifically, as cardiac signals are among the most extensively studied interoceptive modalities, participants completed two established cardioceptive tasks—the heartbeat tracking task and the heartbeat discrimination task—to assess interoceptive performance accuracy, along with associated confidence. In addition, participants also completed the self-rated Multidimensional Assessment of Interoceptive Awareness (MAIA) questionnaire to capture subjective (self-reported), experiential aspects of interoceptive awareness. We then tested whether these interoceptive measures predicted objective (behavioural/task based) and experiential aspects of mental imagery. Participants performed a widely used mental rotation task (Metzler & Shepard, 1974), which indexes the effective deployment of visuospatial imagery. To control for domain-general cognitive demands, we included an n-back working memory task as a non-imagery comparison condition. The subjective experiential aspect of mental imagery was quantified using the self-report Vividness of Visual Imagery Questionnaire-2 (VVIQ-2)^17^. Finally, to examine if any observed associations reflected a broader link between interoception and internal cognitive experiences, we included the self-rated Involuntary Autobiographical Memory Inventory (IAMI), which captures the spontaneous occurrence of imagery-based memories in everyday life.

## Methods

### Participants

A total of 104 participants (77 females, mean age: 22, years, SD = 6.5 years) were recruited from the University of Surrey participant pool and received an Amazon voucher for their participation. All methods were carried out in accordance with relevant guidelines and regulations. All experimental protocols were approved by the University of Surrey Research Ethics Committee. Informed consent was obtained from all participants and/or their legal guardian(s) prior to participation in the study.

### Heartbeat tracking and discrimination tasks

These two cardioceptive tasks were delivered using established procedures^14^ using a validated digital platform that combines a dedicated electrocardiogram (ECG) and R-wave detector^18^ with a tablet computer running bespoke software to deliver instructions and task conditions (HeartRater Clinical: https://www.heartrater.co.uk). This platform is a refinement of methods used in two earlier clinical trials^19,20^ of interoceptive training and was optimised for a third (ongoing) trial^21^. Here, ECG replaced optical (finger or ear pulse photoplethysmography) measures of heartbeat occurrence to mitigate signal/sensor problems common in people with darker skin or poor peripheral circulation. In the heartbeat tracking task (HBT; e.g. Pollatos et al., 2007^22^), participants were instructed: “Without manually checking, can you silently count each heartbeat you feel in your body from the time you hear ‘start’ to when you hear ‘stop’?” This task was repeated six times, with time windows of 25, 30, 35, 40, 45, and 50 s, presented in a randomized order. After each trial, participants entered their responses on a tablet computer. In the heartbeat discrimination task (HBD)^23,24^, participants judged whether a series of ten auditory tones were synchronous with their heartbeat. Each participant received the following instructions: “You will hear ten tones. Please tell me if the tones are in or out of sync with your heartbeat.” This task consisted of 26 trials, with each trial presenting ten tones at 440 Hz with a 100 ms duration, triggered by the sequential heartbeats of the participant. In the synchronous condition, tones were delivered at 250 ms after detection of the ECG R-wave. For the asynchronous condition tones were delivered 550 ms after detection of the R-wave. At the end of each trial, participants responded by stating whether they perceived the tones to be synchronous or asynchronous with their heartbeat. Since tones were always presented at the participant’s own heart rate (either on the heartbeat or time-shifted), participants could not rely on tempo or knowledge of their heart rate to guide responses—only the phase synchrony between tones and heartbeats served as the relevant cue^24^. In both tasks, after each trial, participants immediately rated their confidence in the accuracy of their response. This confidence rating was provided on a tablet-based visual analogue scale (VAS), with “Total guess” on one end and “Total confidence” on the other.

### Mental rotation task (MRT)

This task was adapted from the classic Shepard and Metzler mental rotation experiment and used stimuli from the Mental Rotation Stimulus^25,26^. Each stimulus consisted of 10 white cubes connected in various orientations to form “arms,” presented against a black background. A total of 138 stimuli were selected, all rotating around the x-axis with a full, unobstructed view. The rotation angles (40°, 85°, and 220°) and other stimulus parameters were adapted from Pounder et al. (2022)^26^. The task consisted of one block of 96 trials, with 32 trials per difficulty level. On each trial, participants judged whether two images depicted the same shape in different orientations (48 trials) or completely different shapes (24 trials). The stimuli remained on screen until participants responded, ensuring they had sufficient time to complete the comparison. Before the main task, they completed a practice block of 32 trials to familiarize themselves with the task.

### N-back task

Participants completed a 2-back working memory task as a control outcome to test the specificity of interoception-imagery relationships. This task required continuous monitoring and updating of remembered information: participants viewed a sequence of single-digit numbers (0–9), each presented for 500 ms followed by a 1500 ms fixation cross, and detected when a number matched the one presented two trials earlier (25% of trials were targets). The task consisted of four blocks of 50 trials each, preceded by two practice blocks. Participants provided a confidence rating at the end of each block and were instructed to respond as quickly and accurately as possible using their right index finger.

The N-back task served to test whether associations between interoceptive measures and cognitive outcomes were specific to imagery-related processes or reflected domain-general cognitive effects. If interoceptive measures predict imagery outcomes but not N-back performance, this dissociation would support a selective interoception-imagery relationship rather than general effects on cognitive capacity.

### Questionnaire measures

Self-reported aspects of interoception were measured using the 37-item *Multidimensional Assessment of Interoceptive Awareness (*MAIA-2; Mehling et al., 2018), which captures how individuals perceive and relate to bodily sensations. The scale includes eight subscales: Noticing (awareness of bodily signals), Not-Distracting and Not-Worrying (responses to discomfort), Attention Regulation (ability to focus on bodily sensations), Emotional Awareness (recognizing bodily-emotional links), Self-Regulation (using body awareness to manage distress), Body Listening (seeking insight from bodily cues), and Trusting (feeling safe and at ease in the body). Internal consistency was comparable to the original MAIA-2 validation study (Mehling et al., 2018): Noticing (α = 0.61), Not-Distracting (α = 0.65), Not-Worrying (α = 0.63), Attention Regulation (α = 0.80), Emotional Awareness (α = 0.77), Self-Regulation (α = 0.73), Body Listening (α = 0.76), and Trusting (α = 0.85).

The phenomenology of participants’ mental images was assessed using the *Vividness of Visual Imagery Questionnaire-2* (VVIQ-2)^17^, a 32-item self-report measure. Participants were asked to visualize different scenes, such as a sun rising above the horizon into a hazy sky, and rate how vividly they could picture each one. Responses were given on a 5-point scale, ranging from 1 (“No image at all, only a conceptual understanding”) to 5 (“Perfectly clear and as vivid as real life”), capturing individual differences in imagery strength. The VVIQ2 scale demonstrated good internal consistency (α = 0.85).

Involuntary autobiographical memories were measured using the *Involuntary Autobiographical Memory Inventory (IAMI)*^27^, a 20-item questionnaire designed to assess the frequency and nature of spontaneously occurring autobiographical memories (e.g., “Memories of personal events pop into my mind by themselves—without me consciously trying to remember them”). Each item was rated on a 0–4 scale: Never (0), Once a month or more (1), Once a week or more (2), Once a day or more (3), Once an hour or more (4). IAMI demonstrated good internal consistency (α = 0.87).

### Procedure

Upon arrival at the experimental testing room, and typically approximately 5 min of settling time, participants’ blood pressure and pulse rate were measured twice, the average of which was used in the analyses. The participants then completed the questionnaires (administered in a randomised order via a desktop computer using Qualtrics **(**https://www.qualtrics.com**).** For the performance of the cardiac interoceptive tasks, electrocardiogram (ECG) sensors were placed on the right and left clavicles and the lower left abdomen of each participant to monitor cardiac activity. The participants were seated at a table and instructed to rest their arms on their laps, refrain from crossing their legs, and minimize movement throughout the trials to optimize signal quality. A tablet computer was positioned in front of them, which they used to record responses using their dominant hand. For the performance of the mental rotation and n-back tasks, participants sat facing a computer screen (at a fixed distance of 57 cm). These tasks were presented using Psychopy 2024.2.4 (py3.10) (https://www.psychopy.org/; Peirce et al., 2019)^28^. The interoceptive tasks were conducted first to minimize potential arousal effects of the cognitive tasks on heart-rate measurements otherwise task performance was counterbalanced.

### Data preprocessing

Data were analyzed using the Jamovi software package. All variables were first standardized by converting scores to z-scores. Participants with missing data or chance-level performance on the mental rotation and N-back tasks were excluded on a per-variable basis. Outliers for each variable were removed using the interquartile range (IQR) method, excluding values more than 1.5 times the IQR above the third quartile or below the first quartile.

### Statistical analyses

To test whether interoceptive processes specifically predict mental imagery rather than domain-general cognitive function, we conducted parallel hierarchical regression models for each outcome measure: mental rotation accuracy, mental rotation confidence, VVIQ, involuntary memory, N-back accuracy, and N-back confidence. N-back performance was included as an outcome to test the specificity of interoception-imagery relationships. If interoceptive measures significantly predict imagery-related outcomes but show null associations with N-back working memory performance, this dissociation would demonstrate a selective interoception-imagery relationship rather than domain-general effects of interoceptive processing on cognitive performance. In contrast, significant associations with both imagery-related outcomes and N-back working memory performance would suggest a more general link between interoception and cognitive processing rather than a selective relationship with mental imagery.

For each outcome, we employed a hierarchical linear regression approach with predictors entered in three conceptually distinct blocks:

1. Task-based interoceptive measures: In the first step, behavioural indices of interoceptive ability derived from heart rate tasks were included: HR tracking accuracy, HR tracking confidence, HR discrimination accuracy, and HR discrimination confidence.
2. Autonomic physiological measures: In the second step, we added autonomic nervous system indices—systolic and diastolic blood pressure and resting pulse rate—to capture the influence of physiological arousal on cognition. These were entered after interoceptive accuracy to reflect their role as bottom-up bodily signals shaped by, and subordinate to, central interoceptive inference.
3. Self-reported interoceptive awareness measures: Finally, we included self-reported interoceptive awareness based on the 8 subscales from the Multidimensional Assessment of Interoceptive Awareness (MAIA).

## Results

Descriptive statistics are shown in Table 1.

**Table 1:** Descriptive statistics.

Measure	Mean	SD	Min	Max
Imagery measures				
VVIQ2 (questionnaire range 32–160)	120	19.7	73	158
Mental Rotation Accuracy	0.766	0.128	0.533	1
Mental Rotation Confidence (1–5 scale)	4.27	0.507	3.17	5
Involuntary Memory (questionnaire range 0–80)	45.5	14.7	7.57	79
Control task				
N-back Accuracy	0.803	0.123	0.525	0.985
N-back Confidence (1–5 scale)	3.31	0.801	1.25	4.83
Task-based Interoception				
Heartbeat Tracking Accuracy	0.593	0.235	00.06 .71	0.96
Heartbeat Tracking Confidence (1–10 scale)	5.04	1.9		9.56
Heartbeat Discrimination Accuracy	0.546	0.121	0.31	0.85
Heartbeat Discrimination Confidence (1–10 scale)	5.98	1.2	3.09	8.64
Autonomic Measures				
Systolic BP (mmHg)	110	11.9	85.5	142
Diastolic BP (mmHg)	70.6	9.05	48.5	93.5
Pulse Rate (bpm)	76.4	11	57.5	104
Self-reported interoception				
MAIA Noticing	3.46	0.688	1.5	5
MAIA Not-Distracting	3.22	0.821	1.33	5
MAIA Not-Worrying	2.81	0.616	1	4.33
MAIA Attention Regulation	2.82	0.734	1.29	4.71
MAIA Emotional Awareness	3.58	0.775	1.6	5
MAIA Self-Regulation	2.79	0.851	0.75	5
MAIA Body Listening	2.34	0.897	0.33	4.33
MAIA Trusting	3.46	0.9	1.67	5

Tables 2 and 3 present correlations between interoceptive measures and cognitive outcomes. Task-based interoceptive measures (Table 2) showed selective associations with imagery outcomes. Heartbeat tracking accuracy correlated with VVIQ (*r* = 0.32, *p* < 0.01), while heartbeat discrimination accuracy correlated with mental rotation accuracy (*r* = 0.30, *p* < 0.01). Both interoceptive confidence measures showed moderate-to-strong associations with mental rotation confidence (*r* = 0.30, *p* < 0.01 and *r* = 0.39, *p* < 0.001 for tracking and discrimination confidence, respectively). In contrast, task-based interoceptive measures showed no statistically significant associations with N-back performance.

**Table 2:** Correlations between task-based interoceptive measures and cognitive outcomes.

Task-based interoception	VVIQ	Invol. Mem	MR Acc	MR Conf	*N*-back Acc	*N*-back Conf
HB tracking accuracy	0.32**	− 00.03	0.09	0.01	0.11	−0 0.05
HB tracking confidence	0.16	0.03	− 0.13	0.30**	− 0.08	0.12
HB discrimination accuracy	−0 0.13	− 0.07	0.30**	0.06	0.02	− 0.09
HB discrimination confidence	0.12	− 0.06	−0 0.06	0.39***	0.15	00.18
Cognitive outcomes						
VVIQ	—					
Involuntary memory	0.23*	—				
MR Accuracy	0.06	− 0.15	—			
MR Confidence	0.22	− 0.04	0.27*	—		
N-back Accuracy	0.20	0.01	− 0.05	0.09	—	
N-back Confidence	− 0.08	0.00	−0 0.15	0.03	0.36**	—

*p < .05. **p < .01. ***p < .001. Uncorrected

Self-reported interoceptive awareness (Table 3) revealed distinct patterns across MAIA subscales. MAIA Noticing and Emotional Awareness showed the most consistent associations with imagery outcomes, correlating with both VVIQ (*r*s = 0.24, *p*s < 0.05) and involuntary memory (*r* = 0.31 and 0.34, *p*s < 0.01 and 0.001, respectively). MAIA Trusting correlated positively with VVIQ (*r* = 0.27, *p* < 0.05), while MAIA Not-Worrying showed positive association with involuntary memory (*r* = 0.26, *p* < .01) but a negative correlation with mental rotation confidence (*r* = −0.27, *p* < 0.05). Paralleling the task-based findings, MAIA subscales showed minimal associations with N-back performance, with only Not-Distracting correlating with N-back accuracy (*r* = 0.24, *p* < 0.05) and Self-Regulation with N-back confidence (*r* = 0.24, *p* < 0.05). This pattern of selective associations with imagery but not working memory tasks supports the specificity of the interoception-imagery relationship.

**Table 3:** Correlations between self-reported interoception and cognitive outcomes.

MAIA Subscale	VVIQ	Invol. Mem	MR Acc	MR Conf	*N*-back Acc	*N*-back Conf
Noticing	0.24*	0.31**	– 0 0.06	0.10	0.11	0.18
Not-Distracting	– 0 0.13	0.15	0.11	– 0 0.11	0.24*	0.10
Not-Worrying	– 0 0.01	0.26**	– 0.15	– 0 0.27*	0.17	0.00
Attention Regulation	0.20	0	0.13	– 0 0.05	0.00	0.06
Emotional Awareness	0.24*	0.34***	– 0 0.12	0.12	0.12	– 0.01
Self-Regulation	0.17	– 0.07	– 0 0.11	– 0.03	0.13	0.24*
Body Listening	0.14	0.28**	– 0.17	– 0 0.04	0.06	0.13
Trusting	0 0.27*	– 0.14	– 0.12	– 0 0.03	0.07	0.04

*p < .05. **p < .01. ***p < .001. Uncorrected

Table 4 summarizes the hierarchical regression results across six imagery-related outcomes. VVIQ was significantly predicted by both task-based interoceptive accuracy and self-reported interoceptive awareness, with both Step 1 and Step 3 contributing uniquely to the final model. In contrast, mental rotation accuracy and confidence were predicted only by task-based interoceptive measures (Step 1), with no added explanatory power from physiological or self-report variables. Involuntary memory was significantly predicted only by self-reported interoceptive awareness (Step 3), despite non-significant contributions from earlier steps. No significant effects were found for N-back accuracy or confidence, although the latter showed a trend toward improvement when self-report measures were added. These findings indicate that different forms of mental imagery are supported by distinct interoceptive components, with voluntary and involuntary imagery showing partially dissociable profiles.

**Table 4:** Summary of hierarchical multiple regression analyses.

Outcome variable	Step 1: Task-based Interoception	Step 2: Autonomic measures	Step 3: Self-reported Interoceptive Awareness (8 MAIA subscales)	Total Model: (Adj. *R*²)	Significant model steps
Mental rotation accuracy	R² = 0.177, *p*= .017*	ΔR² = 0.042, *p* = .383	ΔR² = 0.121, *p*= .348	0.143; *p* = .077	Step 1 only
Mental rotation confidence	R² = 0.147, *p*= .043*	ΔR² = 0.017, *p* = .756	ΔR² = 0.168, *p*= .158	0.132; *p* = .092	Step 1 only
Vividness VVIQ	R² = 0.136, *p*= .039*	ΔR² = 0.052, *p* = .257	ΔR² = 0.190, *p*= .043*	0.213; *p* = .012*	Step 1 & Step 3;
Involuntary memory	R² = 0.032, *p*= .664	ΔR² = 0.026, *p* = .594	ΔR² = 0.254, *p*= .010*	0.144; *p* = .048	Step 3
N-back accuracy	R² = 0.011, *p*= .966	ΔR² = 0.088, *p* = .218	ΔR² = 0.121, *p*= .643	–0.080; *p* = .736	No significant steps
N-back confidence	R² = 0.101, *p*= .171	ΔR² = 0.092, *p* = .107	ΔR² = 0.167, *p*= .159	0.161; *p* = .062	No significant steps

Each outcome variable was regressed on three blocks of predictors: (1) Task-based Interoception (HBT and HBD accuracy and confidence), (2) Autonomic measures (systolic BP, diastolic BP, pulse rate), and (3) Self-reported interoceptive awareness (all the 8 MAIA subscales were entered separately). R² = proportion of variance explained. ΔR² = change in variance explained relative to the previous model. Bold p-values indicate statistically significant models (*p* < .05). VVIQ was significantly predicted by both task-based interoception and self-reported interoceptive awareness. Mental rotation accuracy and confidence were predicted only by task-based interoception whereas involuntary memory was predicted only by self-reported interoceptive awareness. No models significantly predicted N-back accuracy.

### Significant predictors: mental rotation accuracy

Across all models, HBD accuracy emerged as a consistent predictor of Mental Rotation accuracy. In the final model, it remained significant (B = 0.403, SE = 0.136, t = 2.97, *p* = 0.005, β = 0.386), indicating that individuals with greater ability to detect their own heartbeats also performed better on the spatial task. No other predictor was statistically significant. Model assumptions were met. Residuals were normally distributed (Shapiro–Wilk = 0.990, *p* = 0.890), independent (Durbin–Watson = 1.75, *p* = .270), and there were no influential outliers (Cook’s Distance Max = 0.157). Multicollinearity was acceptable (all VIFs < 2.36).

### Significant predictors: mental rotation confidence

The MAIA Not-Worrying subscale emerged as a significant negative predictor of confidence on the Mental Rotation task (B = -0.286, SE = 0.0996, t = -2.87, *p* = .006, β = -0.375), suggesting that individuals who report greater worry about bodily sensations tend to be less confident in their spatial decisions. HBD confidence showed a positive but marginal effect (B = 0.312, SE = 0.161, t = 1.94, *p* = .058, β = 0.291). Assumption checks supported the model’s validity: residuals were approximately normally distributed (Shapiro–Wilk = 0.978, *p* = .292), with no strong evidence of autocorrelation (Durbin–Watson = 2.18, *p* = .494) or influential outliers (Cook’s Distance Max = 0.196). Multicollinearity was low to moderate (VIFs < 2).

### Significant individual predictors: Vividness of Visual Imagery (VVIQ)

The significance level of the regression models is shown in Table 4. In terms of the individual predictors, HBT accuracy was a significant positive predictor of VVIQ (B = 0.269, SE = 0.087, t = 3.08, *p* = .003, β = 0.352), indicating that individuals with better heartbeat tracking accuracy reported more vivid visual imagery. Individual differences in resting pulse rate also emerged as a significant predictor (B = 0.276, SE = 0.106, t = 2.61, *p* = .011, β = 0.303), suggesting that higher physiological arousal is associated with increased imagery vividness. Among the self-reported interoceptive awareness subscales, MAIA Noticing (B = 0.186, SE = 0.093, t = 1.99, *p* = .051, β = 0.250), MAIA Emotional Awareness (B = 0.208, SE = 0.106, t = 1.96, *p* = .055, β = 0.253), and MAIA Trusting (B = 0.176, SE = 0.093, t = 1.88, *p* = .065, β = 0.243) each showed positive associations at trend level. Assumption checks supported model validity. Residuals were normally distributed (Shapiro–Wilk = 0.988, *p* = .725), independent (Durbin–Watson = 2.43, *p* = .076), and not influenced by outliers (Cook’s Distance Max = 0.172). Multicollinearity was acceptable, with all VIFs < 2.35.

### Significant individual predictors: Involuntary memory

While Model 3 (see Table 4) was statistically significant, none of the individual predictors reached conventional levels of significance, although MAIA Noticing showed a positive trend (B = 0.266, SE = 0.138, t = 1.92, *p* = .059, β = 0.245), suggesting individuals more attuned to bodily sensations may experience more involuntary memories. Assumption checks indicated normally distributed residuals (Shapiro–Wilk = 0.991, *p* = .892), low autocorrelation (Durbin–Watson = 2.22, *p* = .368), and acceptable multicollinearity (all VIFs ≤ 2.20). No influential outliers were detected (Cook’s Distance Max = 0.083). This suggests that while individual subscale effects were below statistical threshold, the MAIA scales contributed cumulatively to involuntary memory performance.

## Discussion

The aim of this study was to characterize the relationship between (behavioural and self-reported) measures of interoceptive sensitivity and the experience of mental imagery. First, regression analyses indicated that accuracy and confidence in performing a mental rotation task (indexing the capacity to mobilise mental imagery) were linked exclusively to task-based interoceptive measures (HBD performance accuracy and confidence, respectively). In contrast, the reported vividness of visual imagery (VVIQ) was significantly predicted by both task-based interoceptive measures (HBT performance accuracy) as well as self-reported interoceptive awareness (as assessed by MAIA questionnaire). Involuntary autobiographical memory was also significantly predicted only by self-reported interoceptive awareness. For n-back task, none of the models incorporating interoceptive and autonomic measures reached statistical significance.

### Distinct effects of heartbeat tracking (HBT) and heartbeat discrimination (HBD)

A key finding was that the two task-based interoceptive measures (HBT and HBD) were linked to distinct aspects of mental imagery. Better HBT performance accuracy was predictive of increasing vividness of mental imagery whereas HBD predicted performance on a mental rotation task dependent upon the flexible deployment of mental imagery. This dissociation suggests that while interoceptive ability is linked to mental imagery, performance of the HBT and HBD tasks engage different cognitive processes that align respectively with dissociable perceptual (VVIQ) and executive (mental rotation) aspects of mental imagery. The HBT task and rating the d vividness of mental imagery both rely on attention to, and perceptual judgement of, internal representations in the absence of an external reference. In contrast, both the ‘cross-modal’ HBD task and the mental rotation task involve the comparison of an internally generated representation to external information: The HBD task requires individuals to judge whether their heartbeats are synchronous to external stimuli (auditory tones). Similarly, in the mental rotation task, individuals must mentally transform an internal representation of an object and compare its imagined orientation to a target stimulus on the screen.

### Relationship between pulse rate and VVIQ

Pulse rate was also a significant predictor of VVIQ, suggesting that autonomic states may influence imagery vividness, potentially reflecting a broader connection between physiological arousal and internally generated experiences. Pulse rate serves not only as as a direct index of autonomic nervous system (ANS) activity, reflecting the balance between sympathetic and parasympathetic responses, but also as a measure of the feedback (via e.g. baroreceptor activation) of cardiovascular arousal states on brain function. Higher pulse rates are typically associated with greater sympathetic activation, which typically accompanies states of mental alertness and emotional/behavioural engagement. This bidirectional link raises the possibility that the vividness of mental imagery may be amplified by heightened psychophysiological arousal. One explanation is that, though both sympathetic efferent drive and afferent interoceptive pathways, increased autonomic engagement enhances the salience of internally generated representations increasing feelings of reality and immersion. This aligns with research showing that emotional and physiological responses are encoded alongside perceptual experiences, strengthening both recall and imagery^29^. If interoceptive signals, including heart rate fluctuations, provide an embodied foundation for mental imagery, then autonomic activation may amplify the sensory and emotional intensity of internally generated experiences, reinforcing the link between bodily states and cognitive simulations.

### Distinct contributions of task-based and self-reported interoception

While mental rotation performance was solely predicted by task-based interoceptive measures (i.e. Step 1 in regression), the vividness of visual imagery (VVIQ) was additionally predicted by self-reported interoceptive awareness (Step 3 in regression), as assessed by the MAIA questionnaire. This highlights the convergence of two levels of interoception representation towards the construction of the subjective experience of mental imagery. The heartbeat tracking task arguably measures the precision of detecting internal bodily signals, whereas the MAIA focuses on how one pays attention to, emotionally respond to, and regulates one’s bodily experiences. These two measures tap into dissociable processes: HBT performance accuracy, although not immune to long acknowledged top-down influences that constrain over-interpretation, encompasses basic online sensory signal detection, while the MAIA subscales reflect an elaborated integration of interoceptive sensations with past emotional and cognitive experiences. Together, the findings suggest that vivid mental imagery depends on both access to bodily signals and the way in which these signals are integrated sensorially into intentional mental processes.

### Dissociation between voluntary imagery and involuntary autobiographical memory

Notably, the regression analysis indicated that both VVIQ and involuntary autobiographical memory (IAMI) were associated self-reported interoceptive awareness. As both VVIQ and IAMI quantify experiential aspects of mental imagery, this finding supports the view that conscious interoceptive representation provides a foundational substrate for imagery more broadly, whether spontaneously occurring or deliberately constructed. However, only *voluntary* imagery vividness (VVIQ) was also significantly linked to task-based measures of interoceptive accuracy. This pattern suggests that while involuntary imagery may be driven predominantly by bottom-up processes such as spontaneous activation of sensorimotor and interoceptive representations and/or an increased attention devoted to them, voluntary imagery additionally recruits top-down mechanisms that depend on the accurate detection and integration of internal bodily signals to support intentional and vivid mental simulation. Taken together, these findings indicate that subjective interoceptive awareness is a core feature of imagery experiences, yet the volitional construction of imagery relies also on interoceptive precision,

These new observations support the idea that heightened sensitivity to internal bodily signals, such as fluctuations in heart rate, strengthens the connection between physiological states and mental simulations, leading to more vivid mental imagery^7^. Mental imagery, by nature, engages both physiological and emotional responses as it reconstructs past experiences and envisions future events. This mind-body connection is mediated by autonomic responses and interoceptive processes. When encountering a new stimulus, the body undergoes stereotypical changes, including shifts in heart rate and electrodermal activity, which are encoded in memory^29,30^. Later, these physiological responses can be reactivated during recall or imagination, enhancing the immersive quality of imagery. This reactivation is especially evident in emotional imagery, where imagining fear-related scenes can trigger measurable physiological arousal, similar to firsthand experience^31^.

Additionally, these results show that the vividness of mental imagery depends not only on the ability to access internal bodily signals but also on how well individuals integrate these signals with their emotional and cognitive states. Our findings thus support the view that mental imagery is an integrative process, where current and past sensory, emotional, and physiological information dynamically interact, to construct a coherent conscious experience. The identified associations between interoception and imagery suggest that bodily signals provide a foundation for grounding mental simulations in self-referential bodily states. Rather than operating independently, imagery-based cognition is embedded within ongoing physiological processes.

### Limitation and future direction

Several limitations should be considered when interpreting the present findings. First, the heartbeat-based interoceptive tasks used here have been criticised for methodological limitations and sensitivity to non-interoceptive factors^32,33^, and results related to cardiac interoception should therefore be interpreted with caution. In addition, interpretations concerning the role of the autonomic nervous system (ANS) remain partly speculative, as the direct relationship between mental imagery vividness and physiological measures (e.g., heart rate variability) to assess sympathetic–parasympathetic balance were not clear. Moreover, although the mental rotation task was used as a control measure, it should not be considered a direct behavioural equivalent of subjective imagery vividness, as it primarily reflects visual–spatial transformation performance. Future research should employ multimodal physiological assessments, examine the direct modulation of the ANS, and empirically test the proposed top-down and bottom-up mechanisms linking interoception to mental imagery. Nevertheless, our findings highlight the importance of considering the body within cognitive models of imagery and underscore the need for future research to explore how different interoceptive signals—such as heartbeat, respiration, and digestive cues—shape imagery and affective processing.

## Data Availability

The experimental data of this study are available in [https://osf.io/hbdxv/](https:/osf.io/hbdxv) .
